# Editorial: Traditional sporting games and play in physical education: enhancing cultural diversity, emotional well-being, interpersonal relationships and intelligent decisions, volume II

**DOI:** 10.3389/fpsyg.2023.1302853

**Published:** 2023-10-18

**Authors:** Pere Lavega-Burgués, Joao Francisco Magno-Ribas, Miguel Pic

**Affiliations:** ^1^Group of Research in Motor Action (GIAM), National Institute of Physical Education of Catalonia (INEFC), INDEST, University of Lleida, Lleida, Spain; ^2^Grupo de Estudos Praxiologicos—GEP Brasil, Departamento de Desportos Coletivos, Universidade Federal de Santa Maria, Santa Maria, Brazil; ^3^Group of Research in Motor Action (GIAM), University of La Laguna, San Cristóbal de La Laguna, Spain

**Keywords:** traditional games and sports, emotional wellbeing, social inclusion, creativity, motor intelligence, educational contributions, motor praxeology, transdisciplinarity

Lavega-Burgués et al. of this Research Topic, have the pleasure of introducing the work corresponding to the “*Traditional sporting games and play in physical education: enhancing cultural diversity, emotional well-being, interpersonal relationships and intelligent decisions, volume II*”.

This second volume consists of eleven articles that showcase the multifaceted perspective of TSG and play (Figure 1). From a thematic standpoint, this Research Topic explores the effects of TSG on affective, relational, cognitive, organic aspects, and their pedagogical application in physical education. Across various scientific disciplines, the contributions are rooted in motor praxeology, game theory, psychology, pedagogy, sociology, and the Teaching Games for Understanding approach.

**Figure 1:**
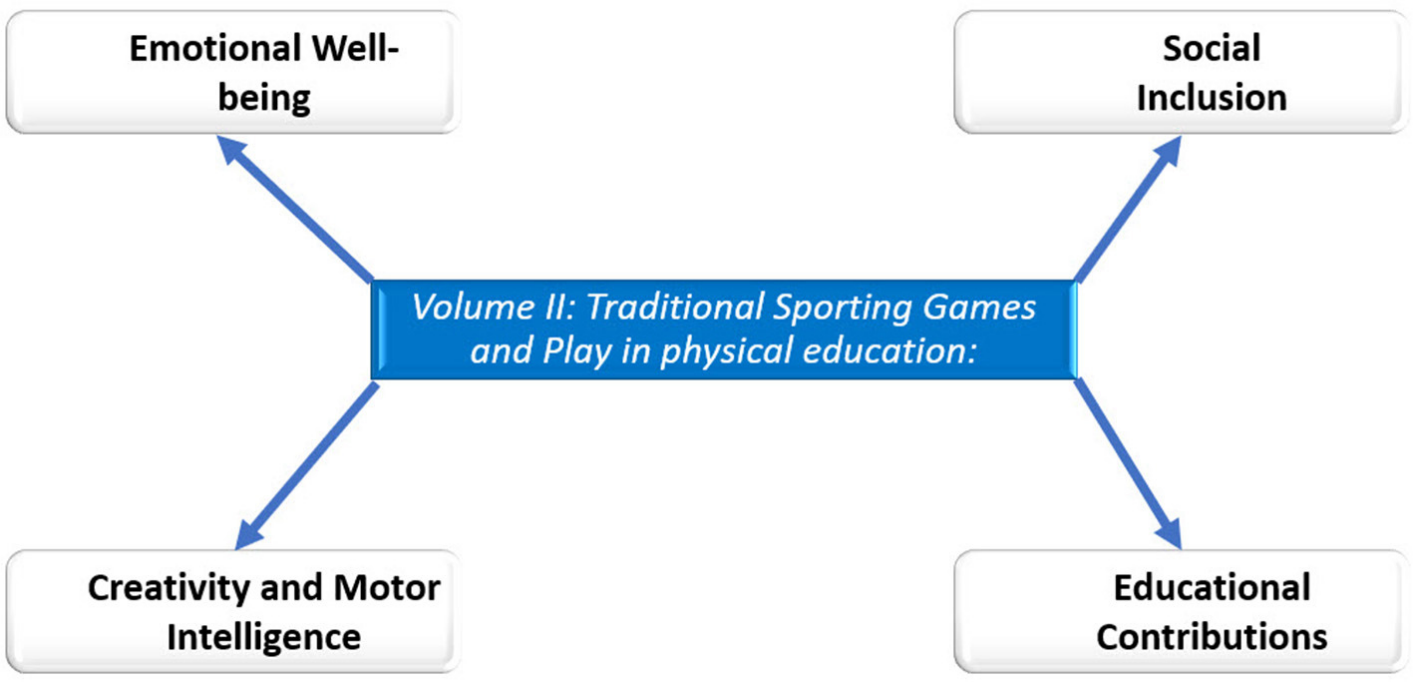
Chapters volume II.

*In the first part*, the *contribution of TSG to the social and emotional well-being* is directly addressed in the first three papers.

In the first article, Moya-Higueras et al. present the research titled “*Traditional Sporting Games as an emotional induction procedure*.” This work, grounded in psychology and motor praxeology, demonstrates the contribution of TSG as a suitable procedure for inducing emotions and studying emotional states in a naturalistic manner, i.e., with ecological validity. Unlike other laboratory procedures, TSG serves as an authentic laboratory for relationships and emotions, where individuals express their emotional states profoundly.

The second research by Dugas and Ben Ali, titled “*Reflection on TSG: the impact of bodily involvement on empathic dimensions*,” investigates bodily engagement and the development of empathy in the practice of TSG. The authors draw on motor praxeology to show that TSG possess an internal logic that triggers the motor involvement of participants, with constant role changes that foster relational empathy within a rich network of social interactions.

This first part concludes with the research of Lavega-Burgués, titled “*Roles, relationships, and motor aggressions: keys to unveiling the emotions of a traditional sporting game*.” The study reveals from a praxeological approach the emotional keys for promoting coexistence and motor aggressiveness education by using a TSG. The employed game confirms the originality of the rules that encompass the internal logic of TSG.

The second part of this volume addresses the *contribution of TSG in the context of social inclusion*. The first article is authored by March-Llanes et al. titled “*Chedoke-McMaster attitudes toward children with handicaps scale for traditional sporting games (CATCH-TSG): initial validation in 7 different languages in adult and young populations*.” This manuscript is one of the collaborative efforts originating from the European Opportunity project to provide a validated questionnaire for examining the effects of TSG in transforming stereotypes related to individuals with intellectual disabilities.

Concurrently, Carter-Thuillier et al. undertake the intercultural research titled “*After-school sports programmes and social inclusion processes in culturally diverse contexts: results of an international multicase study*.” It is evident that the use of TSG from various cultures can facilitate social inclusion processes and interpersonal relationships.

In the third section, two articles delve into the cognitive dimension of Traditional Sports and Games (TSG), highlighting the creativity and motor intelligence they promote. Oboeuf et al. present the study “*Relationships between empathy and creativity in collective games: a comparison between handball and sitting ball*.” Drawing on motor praxeology, the authors compare the effects of a TSG and a sport on creativity and socio-motor empathy, associated with decision-making and cognitive and affective processes.

Subsequently, Schmidt and Ribas draw on motor praxeology to “*Identify and describe the sociomotor sub-roles and the Ludogram of Brazilian jiu-jitsu*.” The article systematizes the decision-making units activated by the internal logic of this TSG, with the aim of facilitating the teaching of motor intelligence in Jiu-Jitsu.

The fourth part consists of four articles that highlight the educational contribution of TSG and play from different perspectives.

Hello et al. grounded in *games theory*, develop the study titled “*The concordance game: a simple tool to estimate breath-hold swimming performance and to teach dynamic apnea*.” It demonstrates the interest of using TSG to improve organic, affective, and decisional aspects present in apnea motor actions.

Ribas et al. undertake the research titled “*How to understand sports and traditional games and how to apply it to physical education. on the “Goal of Game”*.” The authors showcase the use of the “goal of game” concept to classify and promote educational applications of TSG.

Chow et al. present the study titled “*The effect of nonlinear pedagogy on the acquisition of game skills in a territorial game*.” This study demonstrates the benefits of using nonlinear pedagogy, with a focus on Teaching Games for Understanding, to facilitate exploratory learning among students.

Finally, Houser and Kriellaars develop an enriched pedagogical approach with physical literacy. This contribution is aptly titled “‘*Where was this when i was in Physical Education?' Physical literacy enriched pedagogy in a quality physical education context*.”

The *multidimensional perspective offered by this Research Topic* allows for a better understanding of the holistic contribution of physical education and sport. Traditional games, sports, modified games; interventions with schoolchildren, university students, or athletes; traditional pedagogies and innovative pedagogies have all revealed the treasure that accompanies physical activity and sport in general, and TSG in particular.

*In conclusion*, Lavega-Burgués et al. topic editors, of this second volume wish to express their gratitude to all the authors, editors, and reviewers for their magnificent work.

Following the publication of two Research Topic volumes on Traditional Games and Sports, we invite all researchers and educators to join the Worldwide Network of Teachers and Researchers in Traditional Games and Sports: https://jugaje.com/red-profesores/.

Alea Jacta Est!!!

## Author contributions

PL-B: Writing—original draft, Writing—review and editing. JM-R: Writing—original draft, Writing—review and editing. MP: Writing—original draft, Writing—review and editing.

